# Supplementary material for: Nociceptive Cortical Activity Is Dissociated from Nociceptive Behavior in Newborn Human Infants under Stress
Source: Curr Biol. 2017 Dec 18;27(24):3846–3851.e3. doi: 10.1016/j.cub.2017.10.063 (PMC5742634; doi:10.1016/j.cub.2017.10.063)
Supplement: Document S1. Figures S1–S4 [file mmc1.pdf]

Current Biology, Volume *27*

Supplemental Information

Nociceptive Cortical Activity Is Dissociated from Nociceptive Behavior in Newborn Human Infants under Stress

Laura Jones, Lorenzo Fabrizi, Maria Laudiano-Dray, Kimberley Whitehead, Judith Meek, Madeleine Verriotis, and Maria Fitzgerald

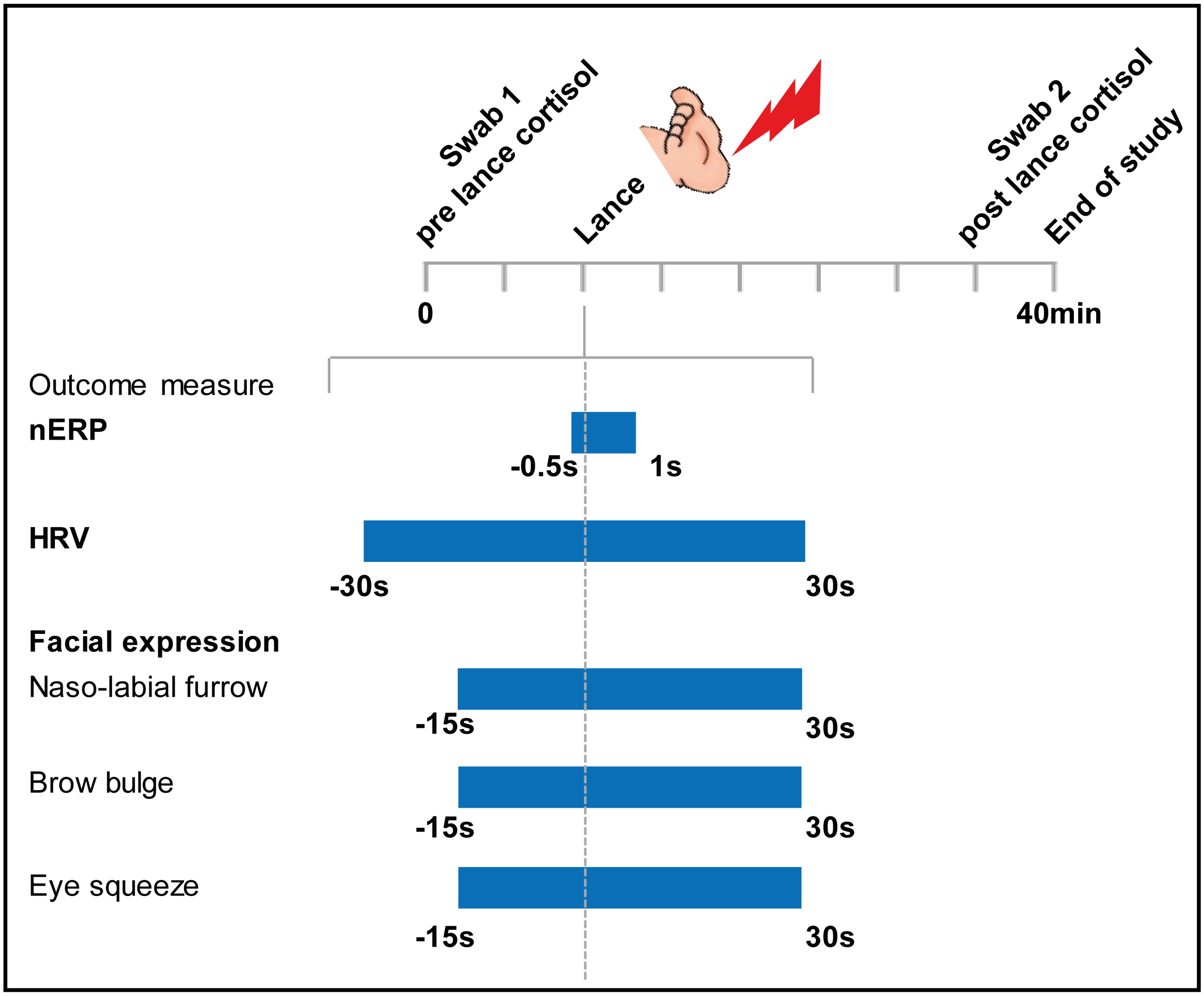

Figure S1. Experimental set-up (Related to Figures 1-4). Timeline of experimental set-up. Saliva samples for cortisol were collected on two occasions, before and after the heel lance. The HRV was calculated from ECG recordings in two 30 second epochs before and after the lance. The nERP was recorded in the 1 sec EEG epoch time-locked to the lance. The facial expression score was calculated from three distinct facial expressions (naso-labial furrow, brow-bulge, an eye squeeze) in the 30sec period after the lance. See also Figure S2 and STAR Methods. nERP = nociceptive event related potential, HRV = heart rate variability

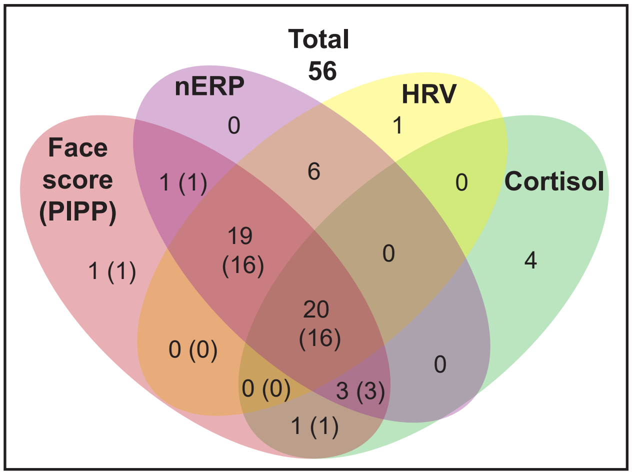

Figure S2. Sample size distribution (Related to Figures 1-4). The distribution of infants with a successful recording of each of the four measures. Of the 56 infants recruited: 49 had EEG recorded, 45 had a facial expression score (40 with PIPP), 46 had an HRV measure, and 29 had sufficient saliva volumes for cortisol analysis. See also Figure S1 and STAR Methods.

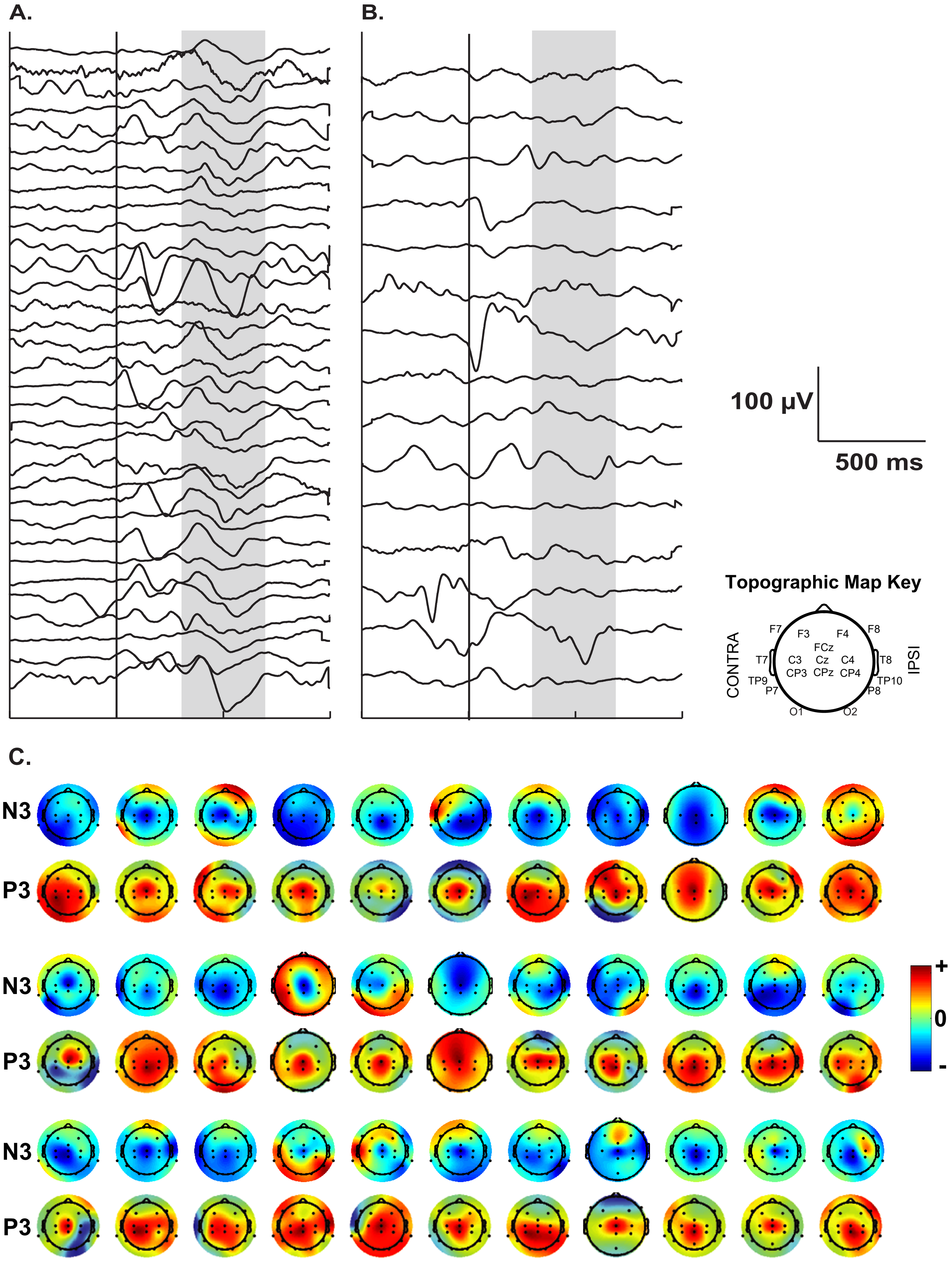

Figure S3. Individual EEG epochs and topographic plots (Related to Figure 2). Waterfall plot of individual EEG epochs recorded at electrode location Cz. The latency window of the nERP is highlighted in grey. (A) All the epochs in which a distinct nERP response was identified following classification from two independent raters. (B) All the epochs in which no distinct nERP was identified. (C) Individual normalized topographic plots of each N3 and P3 peak amplitudes in babies with a response. See also Figure 2.

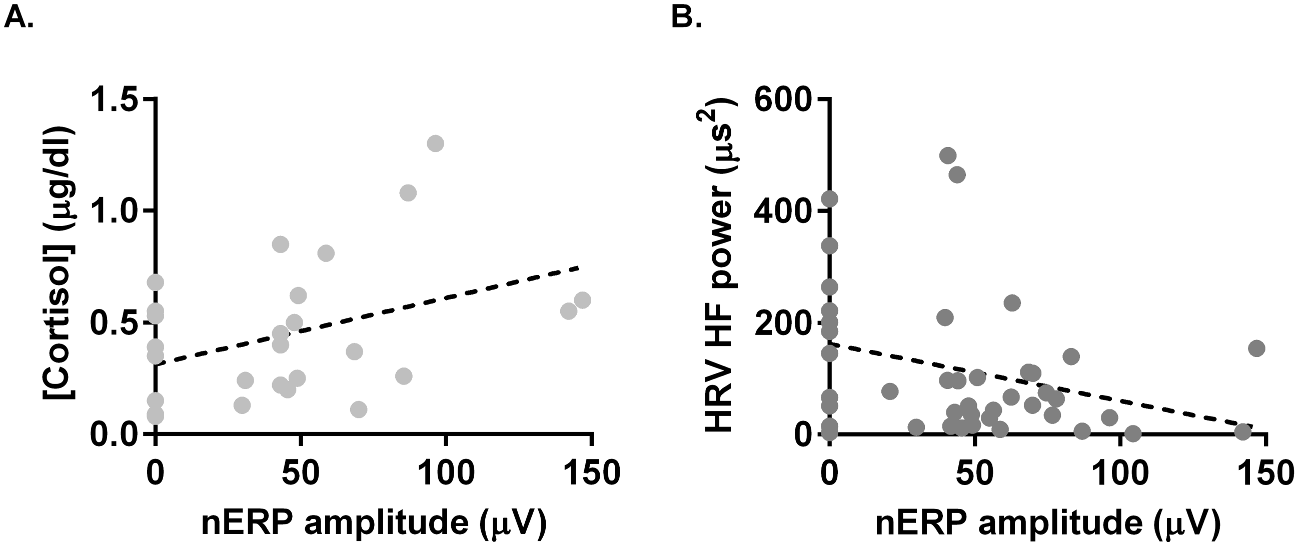

Figure S4. HF HRV power and cortisol concentration are significantly related to nERP amplitude (Related to Figure 4). (A) Correlation between nERP amplitude and cortisol concentration (F(1, 26)=5.36, p=.029, R2=.17). (B) Correlation between nERP amplitude and HRV HF power (F(1, 26)=5.51, p=.027, R2 =.18). See also Figures 3 and 4.
