# Supplementary material for: Nociceptive Cortical Activity Is Dissociated from Nociceptive Behavior in Newborn Human Infants under Stress
Source: Curr Biol. 2017 Dec 18;27(24):3846–3851.e3. doi: 10.1016/j.cub.2017.10.063 (PMC5742634; doi:10.1016/j.cub.2017.10.063)
Supplement: Document S2. Article plus Supplemental Information [file mmc2.pdf]

Report

Current Biology

Nociceptive Cortical Activity Is Dissociated from Nociceptive Behavior in Newborn Human Infants under Stress

Highlights

- Infant pain behavior and nociceptive brain activity are generally correlated
- Stress disrupts the relationship between infant pain brain activity and behavior
- Stress is associated with increased nociceptive brain activity, but not behavior
- Stress is an important factor when assessing infant pain experience

Authors

Laura Jones, Lorenzo Fabrizi, Maria Laudiano-Dray, Kimberley Whitehead, Judith Meek, Madeleine Verriotis, Maria Fitzgerald

Correspondence

m.fitzgerald@ucl.ac.uk

In Brief

Jones et al. show that high physiological stress in infants, measured by cortisol and heart rate variability, is associated with greater cortical pain activity, but not with increased pain behavior. Stress disrupts the relationship between nociceptive brain activity and behavior and is an important extraneous factor when predicting infant pain.

Check for updates

Jones et al., 2017, Current Biology *27*, 3846–3851
December 18, 2017 © 2017 The Authors. Published by Elsevier Ltd.
https://doi.org/10.1016/j.cub.2017.10.063

CellPress

Current Biology
Report

Nociceptive Cortical Activity Is Dissociated from Nociceptive Behavior in Newborn Human Infants under Stress

Laura Jones,[1] Lorenzo Fabrizi,[1] Maria Laudiano-Dray,[1] Kimberley Whitehead,[1] Judith Meek,[2] Madeleine Verriotis,[1,3,4] and Maria Fitzgerald[1,3,5,*]
[1]Department of Neuroscience, Physiology, and Pharmacology, University College London, London WC1E6BT, UK
[2]Elizabeth Garrett Anderson Obstetric Wing, University College London Hospitals, London WC1E6DB, UK
[3]These authors contributed equally
[4]Present address: Developmental Neurosciences Program, University College London Great Ormond Street Institute of Child Health, London WC1N1EH, UK
[5]Lead Contact
*Correspondence: m.fitzgerald@ucl.ac.uk
https://doi.org/10.1016/j.cub.2017.10.063

SUMMARY

Newborn infants display strong nociceptive behavior in response to tissue damaging stimuli, and this is accompanied by nociceptive activity generated in subcortical and cortical areas of the brain [1, 2]. In the absence of verbal report, these nociceptive responses are used as measures of pain sensation in newborn humans, as they are in animals [3, 4]. However, many infants are raised in a physiologically stressful environment, and little is known about the effect of background levels of stress upon their pain responses. In adults, acute physiological stress causes hyperalgesia [5–7], and increased background stress increases pain [8–10], but these data cannot necessarily be extrapolated to infants. Here we have simultaneously measured nociceptive behavior, brain activity, and levels of physiological stress in a sample of 56 newborn human infants aged 36–42 weeks. Salivary cortisol (hypothalamic pituitary axis), heart rate variability (sympathetic adrenal medullary system), EEG event-related potentials (nociceptive cortical activity), and facial expression (behavior) were acquired in individual infants following a clinically required heel lance. We show that infants with higher levels of stress exhibit larger amplitude cortical nociceptive responses, but this is not reflected in their behavior. Furthermore, while nociceptive behavior and cortical activity are normally correlated, this relationship is disrupted in infants with high levels of physiological stress. Brain activity evoked by noxious stimulation is therefore enhanced by stress, but this cannot be deduced from observation of pain behavior. This may be important in the prevention of adverse effects of early repetitive pain on brain development.

RESULTS

Levels of Physiological Stress in Individual Infants

We first measured individual levels of background physiological stress over the test period in our sample. Salivary cortisol concentration and high-frequency heart rate variability (HF HRV) were measured before and after the noxious test procedure. Neither salivary cortisol concentration nor HF HRV was significantly altered by the heel lance (cortisol: pre-lance median: 0.38 μg/dL, range: 0.03–1.74 μg/dL; post-lance median: 0.30 μg/dL, range: 0.07–1.51 μg/dL; $t(27) = 1.73$, $p = 0.094$, 95% CI [0.10, 0.17]) (HF HRV: pre-lance median: 59.98 ms^2, range: 2.23–557.51 ms^2; post-lance median: 90.56 ms^2, range: 0.98–585.66 ms^2; $t(45) = 1.75$, $p = 0.087$, 95% CI [24.47, 41.65]), and cortisol concentration and HF HRV power after the lance were positively correlated with their values preceding the lance (cortisol: $r(28) = 0.39$, $p = 0.039$, 95% CI [0.03, 1.07]; HF HRV power: $r(46) = 0.58$, $p < .001$, 95% CI [0.30, 0.75]). We therefore used the average of the pre- and post-lance values as a measure of stress throughout the test period.

Figure 1 shows the wide range of background stress in our sample population. Salivary cortisol concentration (n = 28, median: 0.38 μg/dL, range: 0.08–1.3 μg/dL) and HF HRV power (n = 46, median: 71.64 ms^2, range: 1.61–499.16 ms^2) were not significantly correlated ($r(20) = -.25$, $p = 0.280$, 95% CI [−0.002, 0.001]). Neither measure was affected by the sleep state or the position of the infant (cortisol: $F(3, 18) = 0.79$, $p = 0.515$; $t(25) = 0.71$, $p = 0.484$; HRV: $F(3, 41) = 1.39$, $p = 0.261$; $t(44) = 0.23$, $p = 0.822$). Salivary cortisol concentration was unaffected by the time since the last feed ($F(2, 25) = 0.44$, $p = 0.650$). See Figures S1 and S2 for experimental design and sample sizes.

Cortical and Behavioral Nociceptive Responses to Heel Lance

We next measured nociceptive behavior and brain activity in response to the time-locked heel lance in individual infants. Behavior was measured using noxious evoked facial grimaces scored from video recordings, and brain activity

 © 2017 The Authors. Published by Elsevier Ltd.
This is an open access article under the CC BY license (http://creativecommons.org/licenses/by/4.0/).

CellPress

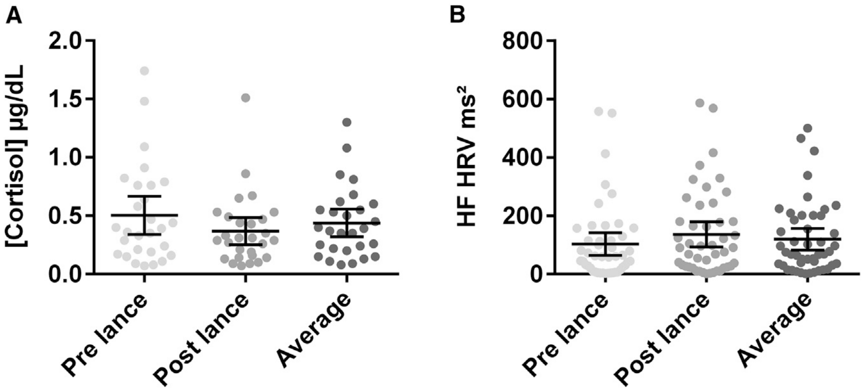

Figure 1. Measures of Physiological Stress in the Sample of Infants

(A and B) Salivary cortisol concentration (A) and heart rate variability high-frequency (HF HRV) power (B) in individual babies before the heel lance (pre-lance), after the heel lance (post-lance), and the average of the two. Horizontal lines represent the mean ± 95% CI. See Figure S1 for experimental design and Figure S2 for sample sizes.

was measured as the amplitude of the nociceptive event-related potential (nERP) recorded with electroencephalography (EEG). The overall pain score (premature infant pain profile [PIPP], a composite behavioral and physiological measure [9]) was also calculated for each baby. The physiological (heart rate and oxygen saturation) response was included for completeness.

The time-locked heel lance evoked a clear nERP with a characteristic N3P3 waveform [11], in 33 infants (67%) (grand average, Figure 2; single subject individual nERPs; Figure S3). The median N3P3 peak-to-peak amplitude of the whole sample, including non-responders, was 44.18 μV (range: 0–146.88 μV, n = 49).

The lance also produced characteristic nociceptive facial behavior in 23 infants (51%, median score: 3, range: 1–9) and a physiological (heart rate and oxygen saturation) response in 40 infants (89%). The PIPP score was calculated (n = 38, median: 4.5, range: 2–17) and indicated that 24 infants exhibited mild to no pain in response to the lance (0–6; 63%), 10 infants moderate pain (7–12; 26%), and 4 infants severe pain (>12; 11%).

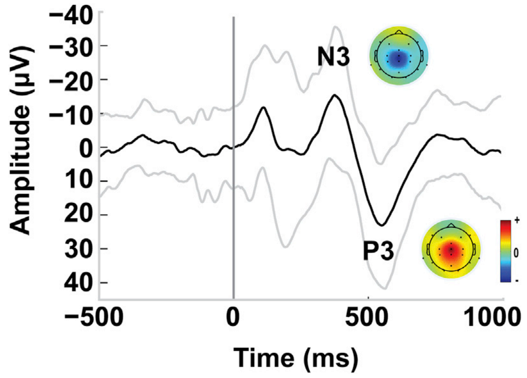

Figure 2. Average Nociceptive Event-Related Potential Waveform Recorded at Cz

Average nociceptive event-related potential (nERP) of 49 infants showing the nociceptive N3P3 wave as recorded at electrode location Cz. The heel lance was applied at time 0. Gray lines represent the standard deviation. Normalized topographic plots are provided for each peak. See Figure S3 for plots of all individual EEG epochs recorded at Cz and individual normalized topographic plots of N3 and P3 peak amplitudes.

All the pain measures were unaffected by the sleep state or position of the baby (nERP: $F(3, 43) = 0.97$, p = 0.417; $t(46) = 0.64$, p = 0.529; facial expression: $F(3, 39) = 0.50$, p = 0.688; $t(43) = -.12$, p = 0.905; HR and O2: $F(3, 32) = 0.95$, p = 0.428; $t(36) = 1.63$, p = 0.112; PIPP: $F(3, 31) = 0.55$, p = 0.649; $t(35) = 0.45$, p = 0.659).

The Relationship between Infant Nociceptive Behavior and Cortical Activity

We next examined the relationship between nociceptive behavior and brain activation following the noxious heel lance in individual infants.

Figure 3A shows a positive relationship between the amplitude of the cortical nERP and the facial behavior, with a trend toward significance (r = 0.28, p = 0.068, 95% CI [−0.02, 0.53]) (Figure 3A). In addition, the nERP amplitude was significantly correlated with the PIPP score ($r(36) = 0.36$, p = 0.033, 95% CI [0.30, 6.87]) (Figure 3B). There was no correlation between nERP amplitude and the physiological score alone (r = 0.19, p = 0.242, 95% CI [−0.13, 0.47]).

Physiological Stress Dissociates Nociceptive Behavior from the Cortical Response

We next tested the influence of background stress upon the relationship between nociceptive behavior and brain activation. On dividing infants into those with low (median: 0.21 μg/dL, range: 0.08–0.37 μg/dL) and high (median: 0.58 μg/dL, range: 0.39–1.3 μg/dL) cortisol levels, linear regression showed that in those infants with a low level of cortisol concentration, there was a significant and strong relationship between facial behavior scores and the nERP amplitude (r(14) = 0.60, p = 0.023, 95% CI [0.10, 0.86]). However, for those infants with a high level of cortisol concentration, this correlation was not significant (r(14) = 0.14, p = 0.630, 95% CI [−0.42, 0.62]) (Figure 3C).

Repeating this analysis, using the top and bottom 25% of cortisol concentrations, produced the same pattern of results. There was no significant correlation between nERP amplitude and facial behavior for the highest 25% = r(7) = −.11, p = 0.822, 95% CI [−0.80, 0.70], but there was significant correlation for the lowest 25% = r(7) = 0.76, p = 0.046, 95% CI [0.02, 0.96].

The Cortical, but Not Behavioral, Nociceptive Response Is Related to the Stress Measures

To explore the reason for this dissociation, we looked at the relationship between stress and nociceptive behavior and cortical activity separately. Figure 4 illustrates how together, HF HRV

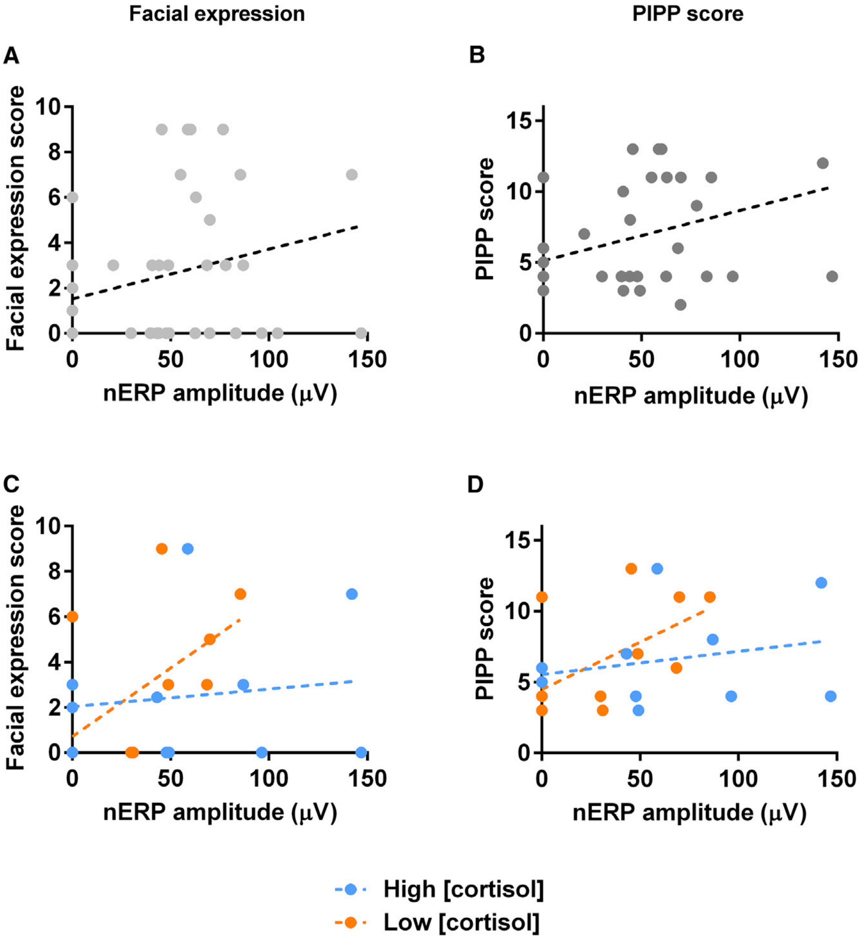

Figure 3. The nERP Amplitude and Facial Expression Score and the nERP Amplitude and Pain Score (PIPP) Are Correlated Only in Infants with Low Cortisol Concentration

(A) A positive relationship between the facial expression score and nERP amplitude shows a trend toward significance (r = 0.28, p = 0.068).

(B) Total PIPP score and nERP amplitude are positively correlated (r = 0.36, p = 0.033). Dots represent measurement from individual subjects and the dashed line the result of the linear regression.

(C) Correlation between nERP amplitude and facial expression score for high (r(14) = 0.14, p = 0.630) and low (r(14) = 0.60, p = 0.023) cortisol concentration.

(D) Correlation between nERP amplitude and PIPP score for high (r(14) = 0.27, p = 0.345) and low (r(14) = 0.57, p = 0.032) cortisol concentration. Orange and blue data points indicate infants with low and high cortisol concentration, respectively. Dotted lines represent the results of the linear regression.

See Figure S4 for correlation between nERP amplitude and cortisol concentration and between nERP amplitude and HRV HF power.

power and cortisol concentration significantly explain 27% of the variance in nERP amplitude (multivariable linear regression: F(2, 25) = 4.57, p = 0.020, R^2 = 0.27, 95% CI [0.02, 0.52]). However, their coefficients were not significantly different from 0 (cortisol: beta = 0.32, t(27) = 1.78, p = 0.087, 95% CI [−6.91, 95.56]; HRV: beta = −.33, t(27) = 1.82, p = 0.081, 95% CI [−.29, 0.02]), which is likely the result of collinearity between the explanatory variables [12].

Consequently, two linear regressions for the nERP amplitude were performed with HRV and cortisol concentration separately. Taken individually, cortisol concentration and HF HRV power had a positive and negative correlation with nERP amplitude, respectively (cortisol: F(1, 26) = 5.36, p = 0.029, R^2 = 0.17, beta = 0.42, 95% CI [6.45, 108.54]; HF HRV: F(1, 26) = 5.51, p = 0.027, R^2 = 0.18, beta = −.42, 95% CI [−.33, −.02]), with cortisol and HRV accounting for a comparable amount of the nERP amplitude variance (17% and 18%, respectively) (Figure S4).

In contrast, cortisol concentration and HF HRV power do not explain a significant amount of the variance in the facial score (F(2, 25) = 0.95, p = 0.401, R^2 = 0.07, 95% CI [−0.09, 0.23]).

DISCUSSION

In this study, we have simultaneously measured stress and pain in individual newborn infants undergoing a noxious stimulus. The aim was to examine the relationship between behavioral and brain measures of infant pain and how physiological stress affects that relationship. We have shown that the magnitude of the nERP generated in the infant brain following a noxious heel lance is linearly related to the magnitude of nociceptive behavior, as measured by the PIPP score and facial expression. However, this relationship is disrupted in infants with high background levels of physiological stress. The nERP measure is larger, suggesting greater activity in the cortical networks responding to noxious input, in the presence of higher physiological stress while pain behavior is not affected. These data indicate the importance of understanding stress levels when measuring the effects of noxious stimulation in non-verbal subjects, as behavior alone will not indicate the extent of brain activation.

Brain and Behavioral Measures of Infant Pain

Behavioral measures are the cornerstone of pain measurement in non-verbal subjects. They are extensively used in animal models [4] and have been used to assess the efficacy of pharmacological and non-pharmacological pain treatments in human infants [13–16]. Under many conditions, behavioral responses to a noxious stimulus are a good reflection of individual pain perception [17] and consistent with this, we have shown a linear correlation between infant nociceptive brain activity and facial pain behavior or PIPP, as reported elsewhere [18, 19]. However, a reduction in pain behavior in infants is not always accompanied by a reduction in pain-related cortical activity [20, 21], leading to questions over the use of behavior alone to assess infant pain [22]. Activity in the brain is not directly linked to autonomic and somatic activity in the body and may relate more closely to

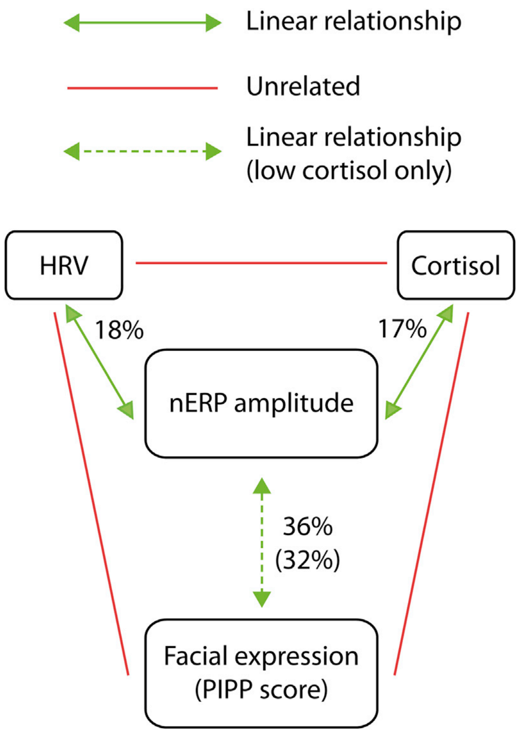

Figure 4. Summary of Relationships between Pain and Stress Measures

There is a significant linear relationship between both measures of physiological stress (cortisol and HRV) and nERP amplitude (green arrows). The significant linear relationship between the facial expression score (and PIPP score) and nERP amplitude is only present in babies with lower levels of cortisol (dashed green arrow). There is no relationship between cortisol and HRV or between these stress measures and facial expression (and PIPP) (red lines).

pain perception. The results here support this, as the two measures differ in their ability to incorporate the level of physiological stress, which is known to increase subjective pain experience in adults.

Reactive and Background Stress

It is important to clarify the difference between acute, reactive stress, and background stress in the context of this study. While we aimed to measure background levels of physiological stress, we noted that the noxious stimulus itself did not cause an acute, reactive stress response. Acute, reactive stress to a defined event or stimulus can follow from coordinated hypothalamic pituitary axis (HPA; cortisol) and sympathetic adrenal medullary (SAM; heart rate variability) activity [23, 24]. However, some individuals demonstrate an uncoordinated relationship between these stress response systems, when one response compensates for another [25–27]. In older infants, the autonomic nervous system and HPA response are not always coordinated at an individual level, in keeping with research on adults [28, 29] and the results reported here.

Previous studies have reported a significant increase in salivary cortisol following a lancet [28], but our finding that blood sampling using a lancet did not elicit a significant acute stress reaction is consistent with other reports [30–32]. The hospital environment can result in high levels of physiological stress, which mask any further increases [33], and cumulative exposure to stressful procedures alters subsequent reactivity [34]. The levels of background stress in our sample varied considerably between individuals, which allowed us to study the relationship between background stress and pain.

The Differential Effects of Stress upon Infant Nociceptive Brain Activity and Behavior

In healthy adults, there is a significant positive correlation between background cortisol levels and pain sensitivity [35]. Experimentally induced background stress or chronic psychosocial stress also results in an increase in pain perception in adult animals [5–7] and humans [8–10], but extrapolating from these data to our infant sample is not straightforward. The effects of stress upon pain pathways are mediated through descending brainstem pathways to the spinal cord [5], and there is evidence of strong descending excitatory drive from serotonergic and other descending pathways over spinal nociceptive circuits in infant and juvenile rats compared to adults [36–38]. This tonic excitation may therefore increase infant pain reactivity to background stress.

However, our data suggest that increase in pain reactivity by background stress in human infants is reflected only in brain activity, possibly through thalamocortical pathways, and not reflected in their motor behavior. The absence of a relationship between background physiological levels of stress and the facial behavioral or the PIPP score is consistent with the report that a second lance 24 hr after the first one leads to attenuated behavior while the cortisol response is unaffected [30]. Indeed, there appears to be no direct correlation between behavior and cortisol or HF HRV in infants [31, 32].

Implications of the Results

Repeated painful and stressful experiences in early life are associated with potentially adverse changes in central nervous system development in both animal models [39] and human infants [40]. The data presented here show that higher levels of background stress in infants are associated with greater noxious evoked activity in the brain, which could contribute to long-term activity-dependent plasticity in the central nervous system. The fact that stress-related brain activity is not accompanied by changes in infant pain behavior means that the influence of stress may escape the attention of caregivers. Furthermore, soothing treatments that reduce pain-related behavioral reactivity may not prevent the increase in brain activity [21, 22].

Little is known about the underlying source of neural activity underlying the nERP [2], and it is not known whether the nERP magnitude is related to the level of pain experienced. The nERP magnitude cannot therefore be simply interpreted as a measure of pain intensity. In healthy adults, the relationship between pain report and ERP amplitude is not direct [41], but ERP magnitudes do reflect levels of central sensitization in pain pathways [42, 43]. Here we use nERP amplitude as a measure of the neural activity in the infant brain evoked by tissue breaking or punctate noxious stimulation [11, 19, 44–46], but not other salient sensory stimuli [47]. While it is possible that the level of physiological stress (as measured by salivary cortisol and HRV) influences the magnitude of the nERP without

influencing the actual pain experience, this does not impact upon the importance of this study. The sensitivity of the nERP to background levels of stress shows that noxious evoked brain activity in individual infants is highly responsive to environmental influences and that the extent of these influences cannot be deduced from behavioral measures alone.

STAR★METHODS

Detailed methods are provided in the online version of this paper and include the following:

- KEY RESOURCES TABLE
- CONTACT FOR REAGENT AND RESOURCE SHARING
- EXPERIMENTAL MODEL AND SUBJECT DETAILS
- METHOD DETAILS
  - Noxious Stimulation
  - Measures of Infant Pain
  - Measures of Infant Stress
- QUANTIFICATION AND STATISTICAL ANALYSIS

SUPPLEMENTAL INFORMATION

Supplemental Information includes four figures and can be found with this article online at https://doi.org/10.1016/j.cub.2017.10.063.

AUTHOR CONTRIBUTIONS

Conceptualization of Study, M.F., L.F., J.M., and M.V.; Experimental Design, M.F., L.F., M.V., L.J., and J.M.; Data Collection, K.W., M.L.-D., and L.J.; Data Analysis, L.J. and K.W.; Data Interpretation, L.J., L.F., M.F., M.V., J.M., M.L.-D., and K.W.; Manuscript Preparation, L.J.; Discussion of Results, Critical Comments, and Revision of Manuscript: L.J., L.F., M.F., M.V., J.M., M.L.-D., and K.W.

ACKNOWLEDGMENTS

This work was funded by the Medical Research Council UK (MR/M006468/1 and MR/L019248/1) and was performed at the National Institute for Health Research/Wellcome UCLH Clinical Research Facility and UCLH Neonatal Unit. The authors thank the families of the infants that participated in this research. L.J. had full access to all the data in the study and takes responsibility for the integrity of the data and the accuracy of the data analysis.

Received: July 29, 2017
Revised: September 20, 2017
Accepted: October 25, 2017
Published: November 30, 2017

REFERENCES

1. Fitzgerald, M. (2005). The development of nociceptive circuits. Nat. Rev. Neurosci. *6*, 507–520.
2. Verriotis, M., Chang, P., Fitzgerald, M., and Fabrizi, L. (2016). The development of the nociceptive brain. Neuroscience *338*, 207–219.
3. Worley, A., Fabrizi, L., Boyd, S., and Slater, R. (2012). Multi-modal pain measurements in infants. J. Neurosci. Methods *205*, 252–257.
4. Mogil, J.S. (2009). Animal models of pain: progress and challenges. Nat. Rev. Neurosci. *10*, 283–294.
5. Jennings, E.M., Okine, B.N., Roche, M., and Finn, D.P. (2014). Stress-induced hyperalgesia. Prog. Neurobiol. *121*, 1–18.
6. Khasar, S.G., Dina, O.A., Green, P.G., and Levine, J.D. (2009). Sound stress-induced long-term enhancement of mechanical hyperalgesia in rats is maintained by sympathoadrenal catecholamines. J. Pain *10*, 1073–1077.
7. Rivat, C., Laboureyras, E., Laulin, J.-P., Le Roy, C., Richebé, P., and Simonnet, G. (2007). Non-nociceptive environmental stress induces hyperalgesia, not analgesia, in pain and opioid-experienced rats. Neuropsychopharmacology *32*, 2217–2228.
8. Crettaz, B., Marziniak, M., Willeke, P., Young, P., Hellhammer, D., Stumpf, A., and Burgmer, M. (2013). Stress-induced allodynia–evidence of increased pain sensitivity in healthy humans and patients with chronic pain after experimentally induced psychosocial stress. PLoS ONE *8*, e69460.
9. Reinhardt, T., Kleindienst, N., Treede, R.-D., Bohus, M., and Schmahl, C. (2013). Individual modulation of pain sensitivity under stress. Pain Med. *14*, 676–685.
10. Saab, C.Y. (2012). Pain-related changes in the brain: diagnostic and therapeutic potentials. Trends Neurosci. *35*, 629–637.
11. Slater, R., Worley, A., Fabrizi, L., Roberts, S., Meek, J., Boyd, S., and Fitzgerald, M. (2010). Evoked potentials generated by noxious stimulation in the human infant brain. Eur. J. Pain *14*, 321–326.
12. Yoo, W., Mayberry, R., Bae, S., Singh, K., Peter He, Q., and Lillard, J.W., Jr. (2014). A study of effects of multicollinearity in the multivariable analysis. Int. J. Appl. Sci. Technol. *4*, 9–19.
13. Codipietro, L., Ceccarelli, M., and Ponzone, A. (2008). Breastfeeding or oral sucrose solution in term neonates receiving heel lance: a randomized, controlled trial. Pediatrics *122*, e716–e721.
14. Johnston, C.C., Stevens, B., Pinelli, J., Gibbins, S., Filion, F., Jack, A., Steele, S., Boyer, K., and Veilleux, A. (2003). Kangaroo care is effective in diminishing pain response in preterm neonates. Arch. Pediatr. Adolesc. Med. *157*, 1084–1088.
15. Murmu, J., Venkatnarayan, K., Thapar, R.K., Shaw, S.C., and Dalal, S.S. (2017). When alternative female kangaroo care is provided by other immediate postpartum mothers, it reduces postprocedural pain in preterm babies more than swaddling. Acta Paediatr. *106*, 411–415.
16. Stevens, B., Yamada, J., Ohlsson, A., Haliburton, S., and Shorkey, A. (2016). Sucrose for analgesia in newborn infants undergoing painful procedures. Cochrane Database Syst. Rev. *7*, CD001069.
17. Sandrini, G., Serrao, M., Rossi, P., Romaniello, A., Cruccu, G., and Willer, J.C. (2005). The lower limb flexion reflex in humans. Prog. Neurobiol. *77*, 353–395.
18. Slater, R., Cantarella, A., Franck, L., Meek, J., and Fitzgerald, M. (2008). How well do clinical pain assessment tools reflect pain in infants? PLoS Med. *5*, e129.
19. Hartley, C., Goksan, S., Poorun, R., Brotherhood, K., Mellado, G.S., Moultrie, F., Rogers, R., Adams, E., and Slater, R. (2015). The relationship between nociceptive brain activity, spinal reflex withdrawal and behaviour in newborn infants. Sci. Rep. *5*, 12519.
20. Ranger, M., Celeste Johnston, C., Rennick, J.E., Limperopoulos, C., Heldt, T., and du Plessis, A.J. (2013). A multidimensional approach to pain assessment in critically ill infants during a painful procedure. Clin. J. Pain *29*, 613–620.
21. Slater, R., Cornelissen, L., Fabrizi, L., Patten, D., Yoxen, J., Worley, A., Boyd, S., Meek, J., and Fitzgerald, M. (2010). Oral sucrose as an analgesic drug for procedural pain in newborn infants: a randomised controlled trial. Lancet *376*, 1225–1232.
22. Pillai Riddell, R., Fitzgerald, M., Slater, R., Stevens, B., Johnston, C., and Campbell-Yeo, M. (2016). Using only behaviours to assess infant pain: a painful compromise? Pain *157*, 1579–1580.
23. Doussard-Roosevelt, J.A., Montgomery, L.A., and Porges, S.W. (2003). Short-term stability of physiological measures in kindergarten children: respiratory sinus arrhythmia, heart period, and cortisol. Dev. Psychobiol. *43*, 230–242.
24. Goldstein, D.S., and Kopin, I.J. (2008). Adrenomedullary, adrenocortical, and sympathoneural responses to stressors: a meta-analysis. Endocr. Regul. *42*, 111–119.

25. McEwen, B.S. (2007). Physiology and neurobiology of stress and adaptation: central role of the brain. Physiol. Rev. *87*, 873–904.

26. Sapolsky, R.M., Romero, L.M., and Munck, A.U. (2000). How do glucocorticoids influence stress responses? Integrating permissive, suppressive, stimulatory, and preparative actions. Endocr. Rev. *21*, 55–89.

27. van Stegeren, A.H., Wolf, O.T., and Kindt, M. (2008). Salivary alpha amylase and cortisol responses to different stress tasks: impact of sex. Int. J. Psychophysiol. *69*, 33–40.

28. Jansen, J., Beijers, R., Riksen-Walraven, M., and de Weerth, C. (2010). Cortisol reactivity in young infants. Psychoneuroendocrinology *35*, 329–338.

29. Waxman, J.A., Pillai Riddell, R.R., Tablon, P., Schmidt, L.A., and Pinhasov, A. (2016). Development of cardiovascular indices of acute pain responding in infants: a systematic review. Pain Res. Manag. *2016*, 8458696.

30. Gunnar, M.R., Hertsgaard, L., Larson, M., and Rigatuso, J. (1991). Cortisol and behavioral responses to repeated stressors in the human newborn. Dev. Psychobiol. *24*, 487–505.

31. Lucas-Thompson, R., Townsend, E.L., Gunnar, M.R., Georgieff, M.K., Guiang, S.F., Ciffuentes, R.F., Lussky, R.C., and Davis, E.P. (2008). Developmental changes in the responses of preterm infants to a painful stressor. Infant Behav. Dev. *31*, 614–623.

32. Ramsay, D., and Lewis, M. (2003). Reactivity and regulation in cortisol and behavioral responses to stress. Child Dev. *74*, 456–464.

33. Bauer, K., Ketteler, J., Hellwig, M., Laurenz, M., and Versmold, H. (2004). Oral glucose before venepuncture relieves neonates of pain, but stress is still evidenced by increase in oxygen consumption, energy expenditure, and heart rate. Pediatr. Res. *55*, 695–700.

34. Grunau, R.E., Holsti, L., Haley, D.W., Oberlander, T., Weinberg, J., Solimano, A., Whitfield, M.F., Fitzgerald, C., and Yu, W. (2005). Neonatal procedural pain exposure predicts lower cortisol and behavioral reactivity in preterm infants in the NICU. Pain *113*, 293–300.

35. Wingenfeld, K., Wolf, S., Kunz, M., Krieg, J.-C., and Lautenbacher, S. (2015). No effects of hydrocortisone and dexamethasone on pain sensitivity in healthy individuals. Eur. J. Pain *19*, 834–841.

36. Hathway, G.J., Koch, S., Low, L., and Fitzgerald, M. (2009). The changing balance of brainstem-spinal cord modulation of pain processing over the first weeks of rat postnatal life. J. Physiol. *587*, 2927–2935.

37. Schwaller, F., Kwok, C., and Fitzgerald, M. (2016). Postnatal maturation of the spinal-bulbo-spinal loop: brainstem control of spinal nociception is independent of sensory input in neonatal rats. Pain *157*, 677–686.

38. Schwaller, F., Kanellopoulos, A.H., and Fitzgerald, M. (2017). The developmental emergence of differential brainstem serotonergic control of the sensory spinal cord. Sci. Rep. *7*, 2215.

39. Schwaller, F., and Fitzgerald, M. (2014). The consequences of pain in early life: injury-induced plasticity in developing pain pathways. Eur. J. Neurosci. *39*, 344–352.

40. Ranger, M., and Grunau, R.E. (2014). Early repetitive pain in preterm infants in relation to the developing brain. Pain Manag. *4*, 57–67.

41. Mouraux, A., and Iannetti, G.D. (2009). Nociceptive laser-evoked brain potentials do not reflect nociceptive-specific neural activity. J. Neurophysiol. *101*, 3258–3269.

42. Iannetti, G.D., Baumgärtner, U., Tracey, I., Treede, R.D., and Magerl, W. (2013). Pinprick-evoked brain potentials: a novel tool to assess central sensitization of nociceptive pathways in humans. J. Neurophysiol. *110*, 1107–1116.

43. Liang, M., Lee, M.C., O'Neill, J., Dickenson, A.H., and Iannetti, G.D. (2016). Brain potentials evoked by intraepidermal electrical stimuli reflect the central sensitization of nociceptive pathways. J. Neurophysiol. *116*, 286–295.

44. Fabrizi, L., Slater, R., Worley, A., Meek, J., Boyd, S., Olhede, S., and Fitzgerald, M. (2011). A shift in sensory processing that enables the developing human brain to discriminate touch from pain. Curr. Biol. *21*, 1552–1558.

45. Verriotis, M., Fabrizi, L., Lee, A., Ledwidge, S., Meek, J., and Fitzgerald, M. (2015). Cortical activity evoked by inoculation needle prick in infants up to one-year old. Pain *156*, 222–230.

46. Verriotis, M., Fabrizi, L., Lee, A., Cooper, R.J., Fitzgerald, M., and Meek, J. (2016). Mapping cortical responses to somatosensory stimuli in human infants with simultaneous near-infrared spectroscopy and event-related potential recording. eNeuro *3*, ENEURO.0026-16.2016.

47. Hartley, C., Duff, E.P., Green, G., Mellado, G.S., Worley, A., Rogers, R., and Slater, R. (2017). Nociceptive brain activity as a measure of analgesic efficacy in infants. Sci. Transl. Med. *9*, eaah6122.

48. Stevens, B., Johnston, C., Petryshen, P., and Taddio, A. (1996). Premature infant pain profile: development and initial validation. Clin. J. Pain *12*, 13–22.

49. Woody, C.D. (1967). Characterization of an adaptive filter for the analysis of variable latency neuroelectric signals. Med. Biol. Eng. *5*, 539–554.

50. Lippman, N., Stein, K.M., and Lerman, B.B. (1994). Comparison of methods for removal of ectopy in measurement of heart rate variability. Am. J. Physiol. *267*, H411–H418.

51. Xhyheri, B., Manfrini, O., Mazzolini, M., Pizzi, C., and Bugiardini, R. (2012). Heart rate variability today. Prog. Cardiovasc. Dis. *55*, 321–331.

52. Mehta, S.K., Super, D.M., Connuck, D., Salvator, A., Singer, L., Fradley, L.G., Harcar-Sevcik, R.A., Kirchner, H.L., and Kaufman, E.S. (2002). Heart rate variability in healthy newborn infants. Am. J. Cardiol. *89*, 50–53.

53. Mörelius, E., He, H.-G., and Shorey, S. (2016). Salivary cortisol reactivity in preterm infants in neonatal intensive care: an integrative review. Int. J. Environ. Res. Public Health *13*, 337.

54. Dempster, A.P., Laird, N.M., and Rubin, D.B. (1977). Maximum likelihood from incomplete data via the EM algorithm. J. R. Stat. Soc. B *39*, 1–38.

STAR★METHODS

KEY RESOURCES TABLE

REAGENT or RESOURCE	SOURCE	IDENTIFIER
Critical Commercial Assays		
Cortisol Enzyme Immunoassay Kit	Salimetrics	1-3002
Software and Algorithms		
MATLAB	MathWorks	R2011b
EEGLab	https://sccn.ucsd.edu/eeglab/index.php	13_5_4b
LabChart HRV module	ADInstrument	MLS310/8
SPSS	IBM Corporation	22
Other		
Neuroscan SynAmps2	Compumedics Neuroscan USA Ltd.	EEG system
Premature Infant Pain Profile (PIPP)	[48]	N/A
Nellcor Oximax	Medtronic	Pulse oximeter

CONTACT FOR REAGENT AND RESOURCE SHARING

Further information and requests for resources should be directed to and will be fulfilled by the Lead Contact, Maria Fitzgerald (m.fitzgerald@ucl.ac.uk).

EXPERIMENTAL MODEL AND SUBJECT DETAILS

Fifty-six healthy term born infants (29 males; 36–42 weeks corrected age, mean 38 weeks + 5 days) aged between 0.5–14 days (3.9 ± 2.4, mean ± SD) were recruited from the postnatal ward and special care baby unit at the Elizabeth Garrett Anderson Obstetric Wing, University College Hospital (UCH). Ethical approval for this study was given by the UCH ethics committee. Informed written parental consent was obtained before each study. The study conformed to the standards set by the Declaration of Helsinki.

METHOD DETAILS

Brain activation, behavioral and physiological responses to a clinically required noxious heel lance and the background salivary cortisol level and heart rate variability (sampled before and after the lance) were recorded (Figure S1). On some occasions, a subset of these measures was acquired because of technical issues such as low sample volumes of saliva (Figure S2).

Noxious Stimulation

The noxious stimulus was a heel lance that was clinically required to collect a blood sample. Lances were never conducted for the sole purpose of the study and were performed by a trained nurse using a disposable lancet. Standard hospital practice was followed during all heel lances. Babies were soothed as and when required. Parents were informed that they could hold their baby if they wished and babies were fed on demand throughout the study. The heel was cleaned and the lancet placed against the heel for at least 30 s prior to the release of the blade. This was done to obtain a baseline period free from other stimulation. The foot was squeezed 30 s after the blade was released to ensure that the cortical, cardiac, and behavioral responses were due to the lancet alone. The release of the blade was time-locked to the ongoing EEG recording using an accelerometer mounted onto the lancet [3]

Measures of Infant Pain

To record the cortical activity following the lance procedure, 21 EEG electrodes were placed on the scalp according to the international 10/20 system. The PIPP was used for the behavioral/physiological composite of infant pain [48].

Electroencephalography

EEG recording: Standard electrode placement, included nineteen electrodes (disposable Ag/AgCl cup electrodes) that were placed according to the modified international 10/20 system at F7, F3, T7, O1, F4, F8, T8, O2, C3, Cz, C4, CPz, CP3, CP4, TP9, TP10, P7, P8, and FCz. Reference and ground electrodes were respectively placed at Fz and FC6/5. Electrode/skin contact impedances were kept to a minimum by gently rubbing the skin with a prepping gel and applying the electrodes with a conductive paste. A soft bonnet was

then secured over the electrodes. EEG activity, from DC to 500cHz, was recorded using the Neuroscan SynAmps2 EEG/EP recording system. Signals were digitised with a sampling rate of 2ckHz and a resolution of 24 bit.

Data pre-processing: Traces were analyzed using EEGLAB and custom-written MATLAB scripts. Raw data were filtered with second-order bidirectional Butterworth bandpass (1–30 Hz) and notch (48–52 Hz) filters. Data were epoched between 0.6 s prior to and 1.1 s following the lance. Baseline correction was carried out using the prestimulus interval. Epochs contaminated with movement artifact (signal exceeding ± 100cμV) were rejected.

Event-related potential (ERP) analysis: Two researchers (LJ and KW) assessed each infant's trial independently and noted the presence or absence of the nERP at the vertex electrode Cz. This event is characterized as a negative-positive waveform occurring between 300–700 ms post-stimulus onset (N3P3) and is not observed following a non-noxious touch stimulus [11]. To compensate for differences in the latency of the ERP, epochs were aligned by Woody filtering [49] between 300−700 ms post stimulus (maximum jitter of −50 to +50 ms). Peaks were identified if distinct from the baseline and were then cross-checked with the raw EEG trace to ensure peaks were not part of any ongoing EEG activity. This resulted in a substantial agreement between the two raters (Cohen's $k = 0.80$ (95% CI, 0.63 to 0.97), $p < 0.001$). Cases in which there was a disagreement were resolved in a consensus session. When the nERP was considered present, its amplitude was measured as the difference between the positive and negative peak amplitudes (N3 – P3). Sixteen babies (33%) did not exhibit the nERP and were given an amplitude of 0.

Premature Infant Pain Profile

Video recording and pulse oximeter: A PIPP score was calculated for each test occasion combining behavioral and physiological measures [48]. Infant facial behavior was recorded on video [3]. Beat-by-beat blood oxygenation and heart rate were monitored with a pulse oximeter (Nellcor Oximax) using a flexible infant probe wrapped around the lateral aspect of the unlanced foot and held in place using a soft Velcro strap.

PIPP scoring: Three facial features were assessed during a 15 s pre-lance baseline period and 30 s post-lance (nasolabial furrow, eye squeeze, and brow bulge). Scores were determined by the percentage of time each expression was exhibited during the 30 s period post-lance. The overall baby's behavioral state at baseline was also assessed, and classified as either quiet awake, active awake, quiet sleep or active sleep.

For the physiological aspects of the PIPP measure, the pulse rate and blood saturation scores were determined by the difference between the baseline pre-lance averages (15 s) and the min/max levels occurring within 30 s post-lance.

The maximum PIPP score, obtained by combining the behavioral and physiological scores, the behavioral state score, and a gestational age score, is 21 with 0–6 indicating minimal/no pain, 7–12 slight/moderate pain, and >12 severe pain.

Measures of Infant Stress

Heart Rate Variability

Electrocardiogram (ECG) was recorded using the same Neuroscan SynAmps2 as for the EEG. Two additional electrodes were placed on the infant's chest in order to record a lead I ECG throughout the study. As with EEG, the recording was time locked to the triggering of the lancet.

HRV analysis: Heart rate variability was measured on two 30 s epochs before (pre-lance) and after (post-lance) the stimulus. Data were bandpass filtered between 1-50Hz before automated beat detection was performed using LabChart HRV software (ADInstruments, Spechbach, Germany). All data were visually inspected and missing beats were manually added if necessary. RR intervals were then obtained by calculating the time between each successive beat. RR intervals were removed if confounded by movement artifact or ectopic beats [50]. A maximum of 4 s were deleted from a trial ($n = 3$). High-frequency (HF, 0.15–1.1 Hz) variation of the beat-to-beat interval in the pre- and post-lance segments was computed and a power spectrum plot was generated using the Lomb Periodogram. HF power reflects frequent beat-to-beat changes in the heart rate, which is driven by respiration (respiratory sinus arrhythmia) when the parasympathetic nervous system (PNS) has greater control. Accordingly, lower HF power indicates the withdrawal of the PNS during the activation of the SAM system, and therefore more physiological stress [51]. HRV HF power values were within the normal ranges as published elsewhere [52].

Salivary Cortisol

Sample collection: Salivary cortisol concentration was measured in two saliva samples collected 10 minutes before the lance and 25 minutes after [32]. An additional swab was used approximately 10 min before the start of the study in order to remove any excess saliva and milk from the mouth. All saliva samples were collected by a research nurse using a cotton swab. The swab, which is 9cm long, was held at one end by the nurse and gently placed into the infant's mouth for up to 5 minutes. If infants became restless at any point the swab was removed. The swabs were then frozen at −20°C until ready for analysis.

Cortisol analysis: Samples were assayed in duplicate when possible at a Salimetrics lab, using an enzyme immunoassay that has a lower limit of sensitivity of 0.007μg/dL and a standard curve range from 0.012 to 3.0 μg/dL. The average intra- and inter-assay coefficients of variation were low (4.4% and 7.6%, respectively). Cortisol concentrations measured were in line with previous research [30, 53].

QUANTIFICATION AND STATISTICAL ANALYSIS

In order to identify any significant stress response, we compared the pre- and post-lance values for cortisol concentration and HRV HF power using Student's t tests. To establish the baseline stress levels throughout the lance procedure, we averaged the pre- and

post-lance values for both cortisol concentration and HRV HF power. We then assessed the relationship between the two baseline measures of stress (cortisol and HRV) and the measures of pain (nERP and PIPP) independently using linear correlations.

Due to the limited number of babies that had a valid cortisol measure as well as all other measures, missing cases were replaced for further analysis that involved cortisol as a variable. First it was confirmed that data were missing at random using the MCAR test (χ^2 (18) = 12.08, ns), and then missing data were replaced using the expectation maximization method [54]. For specific linear correlations and multivariate linear regression, all babies with available cortisol data were used and missing values for the nERP, PIPP, and HRV were subsequently replaced (5, 8, and 8 cases, respectively). For regressions that did not involve cortisol as a variable, only original data were used.

To assess the effect of cortisol levels on the relationship between the nERP and PIPP score, the data were split according to the cortisol concentrations (highest 50% and lowest 50%, 14 babies in each group). The significance of the correlation between the two measures of pain was then tested separately in the two groups. This analysis was repeated using the top and bottom 25% of cortisol concentrations (7 babies in each group).

We explored the relationship between the pain and stress measures with multivariate linear regression modeling using the Enter Method. A separate regression was conducted for nERP amplitude and PIPP score with cortisol concentration and HRV HF power as explanatory variables. All statistical analyses were conducted in SPSS (IBM Corp, Version 22). Significance was set at $p < .05$.

Current Biology, Volume *27*

Supplemental Information

Nociceptive Cortical Activity Is Dissociated from Nociceptive Behavior in Newborn Human Infants under Stress

Laura Jones, Lorenzo Fabrizi, Maria Laudiano-Dray, Kimberley Whitehead, Judith Meek, Madeleine Verriotis, and Maria Fitzgerald

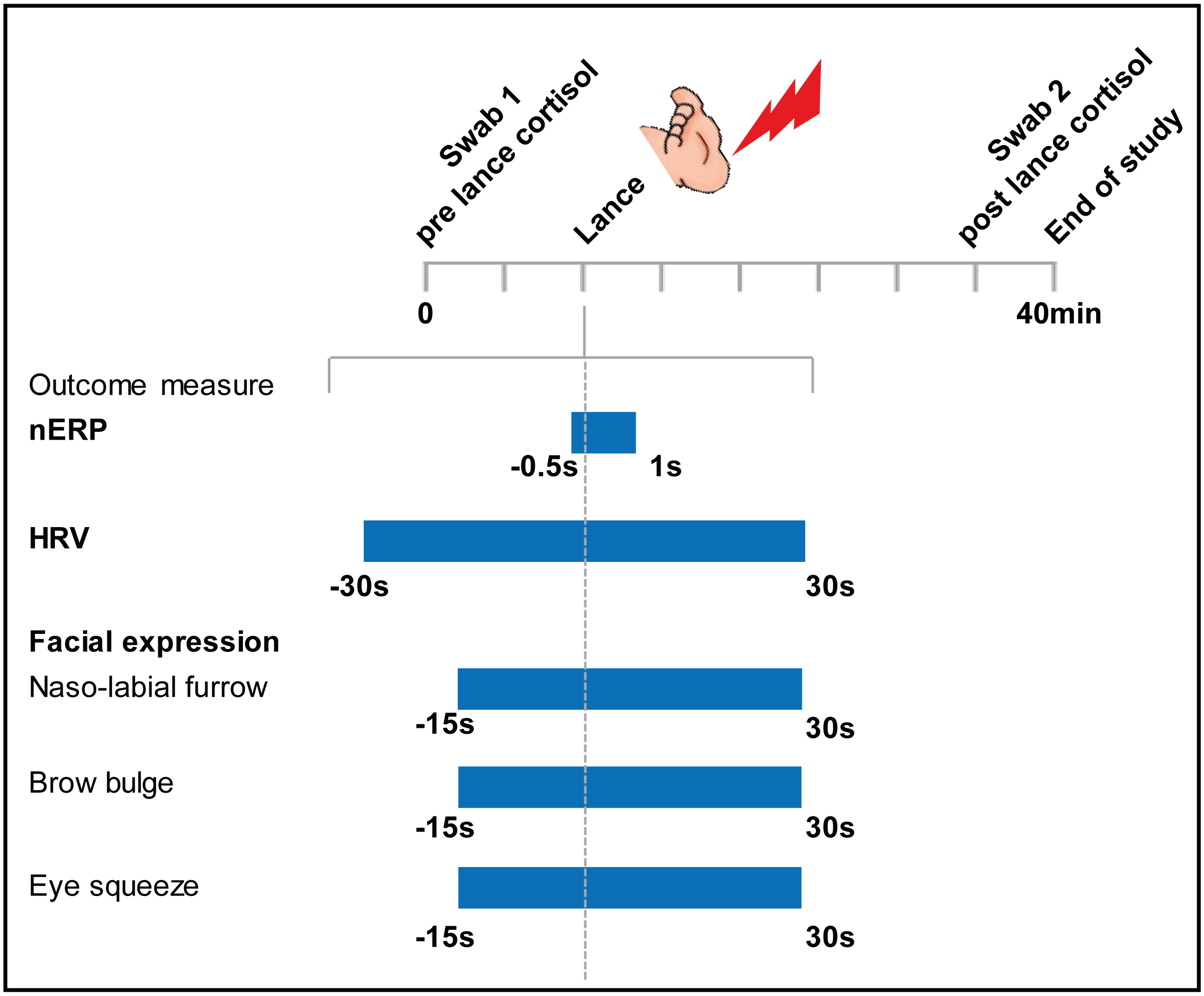

Figure S1. Experimental set-up (Related to Figures 1-4). Timeline of experimental set-up. Saliva samples for cortisol were collected on two occasions, before and after the heel lance. The HRV was calculated from ECG recordings in two 30 second epochs before and after the lance. The nERP was recorded in the 1 sec EEG epoch time-locked to the lance. The facial expression score was calculated from three distinct facial expressions (naso-labial furrow, brow-bulge, an eye squeeze) in the 30sec period after the lance. See also Figure S2 and STAR Methods. nERP = nociceptive event related potential, HRV = heart rate variability

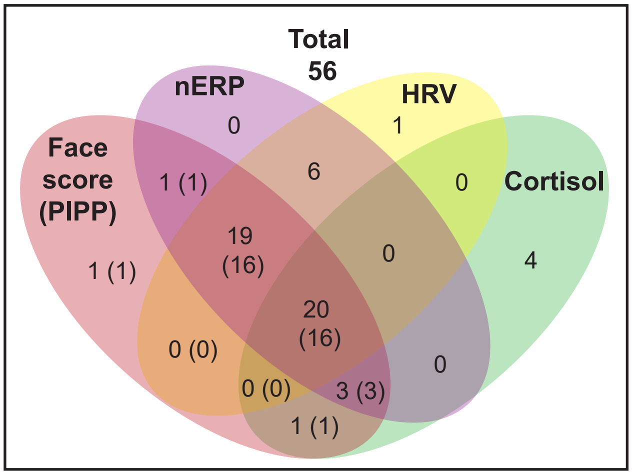

Figure S2. Sample size distribution (Related to Figures 1-4). The distribution of infants with a successful recording of each of the four measures. Of the 56 infants recruited: 49 had EEG recorded, 45 had a facial expression score (40 with PIPP), 46 had an HRV measure, and 29 had sufficient saliva volumes for cortisol analysis. See also Figure S1 and STAR Methods.

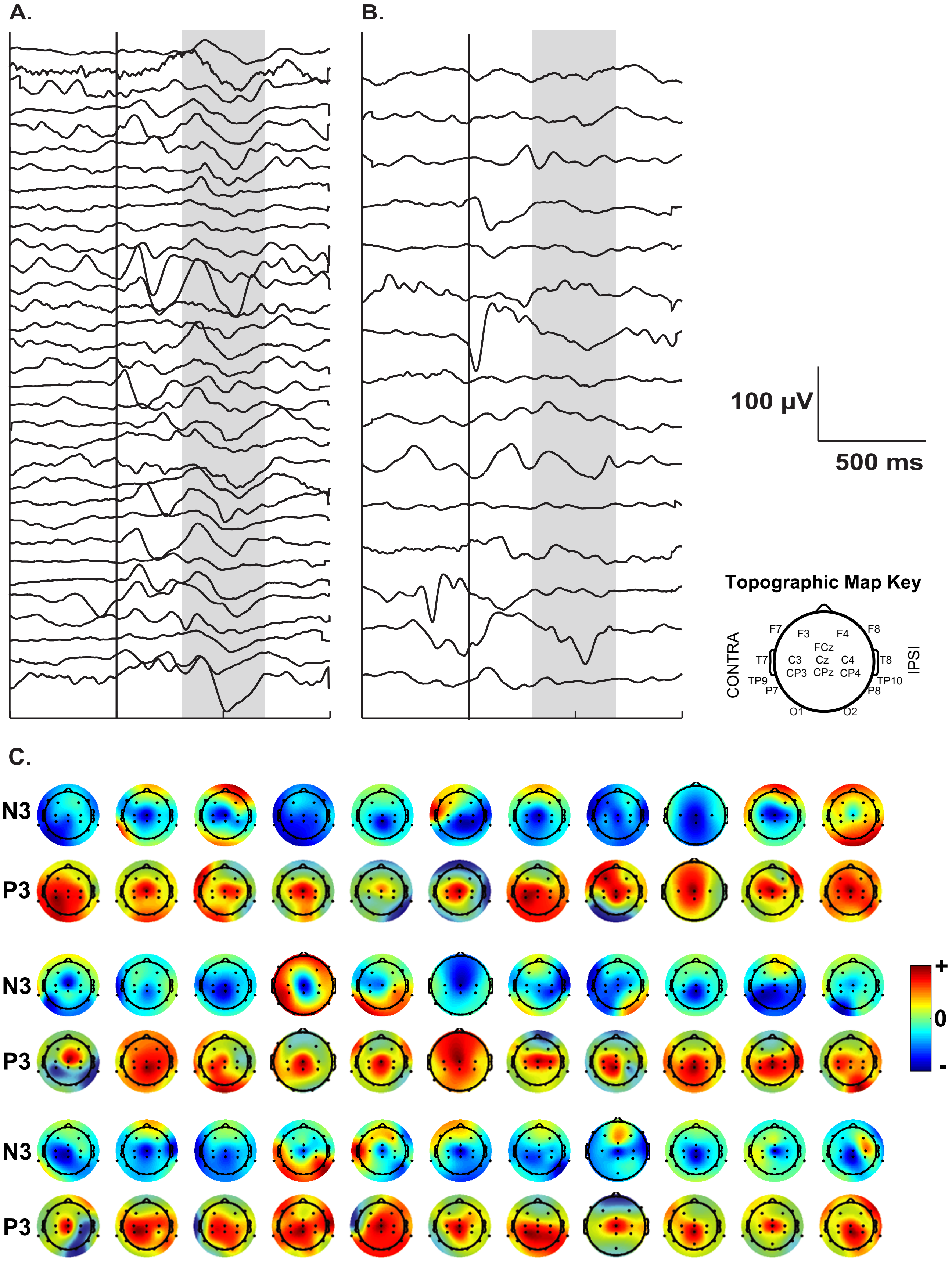

Figure S3. Individual EEG epochs and topographic plots (Related to Figure 2). Waterfall plot of individual EEG epochs recorded at electrode location Cz. The latency window of the nERP is highlighted in grey. (A) All the epochs in which a distinct nERP response was identified following classification from two independent raters. (B) All the epochs in which no distinct nERP was identified. (C) Individual normalized topographic plots of each N3 and P3 peak amplitudes in babies with a response. See also Figure 2.

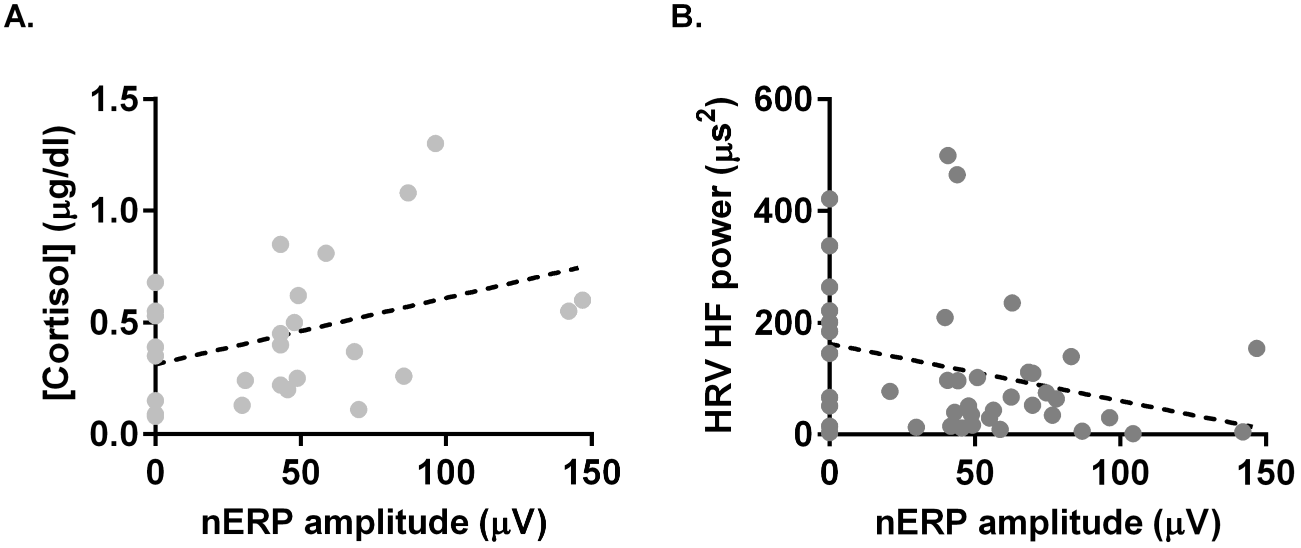

Figure S4. HF HRV power and cortisol concentration are significantly related to nERP amplitude (Related to Figure 4). (A) Correlation between nERP amplitude and cortisol concentration (F(1, 26)=5.36, p=.029, R2=.17). (B) Correlation between nERP amplitude and HRV HF power (F(1, 26)=5.51, p=.027, R2 =.18). See also Figures 3 and 4.
